# Individual Differences in Attention and Intelligence: A United Cognitive/Psychometric Approach

**DOI:** 10.3390/jintelligence9030034

**Published:** 2021-07-02

**Authors:** Andrew R. A. Conway, Kristof Kovacs, Han Hao, Kevin P. Rosales, Jean-Paul Snijder

**Affiliations:** 1 Division of Behavioral & Organizational Sciences, Claremont Graduate University, Claremont, CA 91711, USA; han.hao@cgu.edu (H.H.); kevin.rosales@cgu.edu (K.P.R.); Jean-paul.snijder@cgu.edu (J.-P.S.); 2 Institute of Psychology, ELTE Eotvos Lorand University, 1075 Budapest, Hungary; kovacs.kristof@ppk.elte.hu

**Keywords:** intelligence, attention, working memory

## Abstract

Process overlap theory (POT) is a new theoretical framework designed to account for the general factor of intelligence (*g*). According to POT, *g* does not reflect a general cognitive ability. Instead, *g* is the result of multiple domain-general executive attention processes and multiple domain-specific processes that are sampled in an overlapping manner across a battery of intelligence tests. POT explains several benchmark findings on human intelligence. However, the precise nature of the executive attention processes underlying *g* remains unclear. In the current paper, we discuss challenges associated with building a theory of individual differences in attention and intelligence. We argue that the conflation of psychological theories and statistical models, as well as problematic inferences based on latent variables, impedes research progress and prevents theory building. Two studies designed to illustrate the unique features of POT relative to previous approaches are presented. In Study 1, a simulation is presented to illustrate precisely how POT accounts for the relationship between executive attention processes and *g*. In Study 2, three datasets from previous studies are reanalyzed (*N* = 243, *N* = 234, *N* = 945) and reveal a discrepancy between the POT simulated model and the unity/diversity model of executive function. We suggest that this discrepancy is largely due to methodological problems in previous studies but also reflects different goals of research on individual differences in attention. The unity/diversity model is designed to facilitate research on executive function and dysfunction associated with cognitive and neural development and disease. POT is uniquely suited to guide and facilitate research on individual differences in cognitive ability and the investigation of executive attention processes underlying *g*.

## 1. Introduction

Kovacs and Conway (2016a) proposed a novel account of the general factor of intelligence (*g*) called process overlap theory (POT). According to POT, *g* is the result of multiple domain-general executive attention processes and multiple domain-specific processes that are sampled in an overlapping manner across a battery of intelligence tests (or cognitive tasks). By this account, *g* is a composite of several broad cognitive abilities and, therefore, does not represent a psychological attribute or a general mental ability. The hypothesized sampling procedure in POT is motivated by cognitive psychology research on working memory and is formally expressed in a multidimensional IRT model, POT-I (Kan et al. 2016; Kovacs and Conway 2016b). Hence, POT is more than a modern version of the original—and purely mathematical—sampling model (Thomson 1916); it is a united psychometric/cognitive theoretical framework that integrates experimental and differential approaches to the study of cognitive abilities and intelligence (Cronbach 1957). This integrated approach allows for new investigations into the nature of intelligence, as evidenced by the current Special Issue. It also allows for an account of well-established findings on intelligence. For example, POT explains the hierarchical psychometric structure, ability differentiation, the worst performance rule, and the strong correlations among *g*, fluid reasoning, and working memory capacity (Kovacs and Conway 2016a, 2019a, 2019b; Kovacs et al. 2019).

A central claim of POT is that domain-general executive attention processes play a critical role in intelligence, acting as a central bottleneck on task performance and a constraint on development of domain-specific cognitive abilities. However, Kovacs and Conway (2016a) did not provide a specific account of the executive attention processes; what are they, how are they related, how do they constrain performance? To be fair, Kovacs and Conway (2016a) did mention some executive attention processes identified by Engle et al.’s research on working memory, for example, goal maintenance, selective attention (filtering), interference control (retrieval), and inhibition of dominant or prepotent responses. They also mentioned processes identified by Miyake et al.’s research on the unity/diversity model of executive function, such as shifting between mental sets, updating and monitoring working memory representations, and inhibition. Nevertheless, it is not clear how these processes are related, how they are measured, or what role they play in intelligence.

Furthermore, POT’s identification of executive attention processes as domain-general was indirect, from both a theoretical and an operational perspective. Theoretically, Kovacs and Conway (2016a) cited ample evidence from research on working memory to suggest that the strong and well-established correlation between working memory capacity and intelligence is driven by executive attention (Engle 2002). Relatedly, they relied on a large number of studies that pointed to the neural substrates of executive functions as ones that are involved in diverse cognitive activity (Assem et al. 2020; Duncan and Owen 2000; Miyake et al. 2000). Operationally, they focused on latent variable studies that defined executive function as what working memory tasks measure above and beyond storage and retrieval, instead of focusing on manifest attention tasks that tend to have low reliability (Hedge et al. 2018). Overall, Kovacs and Conway (2016a) highlighted the role of the frontal/executive system but treated it as a black box.

This is an obvious limitation of the theory but it was an intentional decision to remain agnostic with respect to the specific executive attention processes underlying *g*. The current literature on individual differences in attention is extremely diverse in terms of theory, tasks, measures, and research goals. The empirical evidence does not support any one theory, task paradigm, or approach. In our view, it would be a mistake to speculate at this point and adopt one view over another. For example, Engle et al. consider “attentional control” to be a broad ability reflected by a unitary factor, whereas Miyake et al. prefer a three-factor model with distinct “executive functions”. The Engle approach is motivated by cognitive psychology research on working memory and studies of individual differences in typical populations. In contrast, the Miyake et al. approach is motivated by neuroscience research on the prefrontal cortex and neuropsychological assessments of frontal damage and disease. These are fundamentally different approaches, with different goals, and they are, thus, difficult to reconcile. Those are just two examples. Consider two more: Oberauer’s model of working memory (Oberauer 2009) and Braver’s dual mechanisms of control framework (Braver 2012). Oberauer’s model is incompatible with Engle’s view of working memory, while Braver’s approach fractionates cognitive control into two different modes (proactive and reactive), and it is not clear how (or if) this maps onto constructs from other models.

To make matters worse, different subdisciplines use different terms for attention and executive processes, for example, executive attention, executive functions, controlled attention, cognitive control, and attention control. Moreover, to make matters even worse, classic attention tasks like the Stroop and flanker are designed from an experimental perspective and, therefore, are not well suited for measuring individual differences, as evidenced by weak psychometric properties (Hedge et al. 2018). Lastly, in Baddeley’s original model of working memory, the “central executive” is one of the components of working memory; however, in some recent models of attention, the exact opposite is true; working memory is considered one of several executive functions. This is a bewildering set of problems. 

In the current paper, we begin to tackle these problems and focus on three specific aims. First, we discuss barriers to “theory building” in the context of individual differences research on attention and discuss two fundamental problems in the literature: (a) the conflation of theoretical and statistical models, and (b) problematic inferences from latent variable models (Fried 2020). Second, we clarify our own use of the following terms: executive processes, executive functions, attentional control, and cognitive control. Third, we empirically demonstrate advantages of the POT framework for investigating individual differences in attention and intelligence. 

We begin with an overview of sampling models of *g* and a brief review of POT. We then address the fundamental issues mentioned above, which are reflected in studies by Engle et al. and Miyake et al. This allows us to clarify our terminology. Lastly, we present two studies designed to contrast POT with the unity/diversity model of executive function (Miyake et al. 2000).

### 1.1. Sampling Models of g

The positive manifold, which refers to the observed pattern of all positive correlations among different cognitive tests, can be statistically accounted for by a general factor or Spearman’s *g* (Spearman 1904). In more recent models of intelligence, such as the Cattell–Horn–Carroll model, *g* is a higher-order factor and accounts for the observed covariance among broad cognitive ability factors, such as verbal and spatial ability (Cattell 1963; McGrew 2005). The *g* factor typically accounts for 40–60% of the variance in intelligence tests (Deary et al. 2010; Jensen 1998) and is predictive of a wide range of life outcomes, including academic achievement (O’Connell 2018; Roth et al. 2015), job performance and income (Gensowski 2018; Schmidt and Hunter 2004), and even health and longevity (Calvin et al. 2011; Deary et al. 2019).

The most common interpretation of *g* is that it represents a general mechanism that causes performance on different ability tests to be correlated: either a general mental ability (Gottfredson 1997) or a general parameter of cognition that affects all cognitive activity. Thus, in most psychometric models of intelligence, *g* is specified as a “reflective” latent variable, implying that *g* is a measure of a “real” attribute that varies across people. In other words, *g* is a latent variable that is thought to “reflect” a psychological attribute, which is a *cause of performance of cognitive tests*. In contrast, if *g* were a “formative” latent variable, then it would be considered to be a *consequence of performance on cognitive tests.*

Furthermore, *g* has been equated with various constructs, such as information processing speed (Jensen 1998), working memory capacity (Kyllonen and Christal 1990), attentional control (Engle 2002), or mitochondrial functioning (Geary 2018). In cognitive neuroscience, the dorsal-lateral region of the prefrontal cortex is often claimed to be the neural basis of *g* (Duncan 2010). In industrial–organizational psychology, *g* is equated with “the ability to learn” (Schmidt and Hunter 2004). In school psychology, *g* is often interpreted as a general ability in the aptitude/achievement discrepancy approach to the diagnosis of learning disabilities.

An alternative interpretation of *g* is that it is the consequence, not the cause, of the correlations between various cognitive abilities. According to the sampling approach, *g* does not represent a general cognitive ability. Instead, there are thought to be multiple cognitive processes involved in the performance of intelligence tests, and these processes are sampled across a battery of tests. The covariance among intelligence tests—and, ultimately, the *g* factor—is caused by an overlap of processes tapped by different tests. According to this view, *g* does not represent a “real” psychological attribute. 

This kind of interpretation originates in the work of Godfrey Thomson, who in 1916 proposed a direct challenge to Spearman’s view (Thomson 1916). Thomson argued that there are a large number of mental “bonds”, and a sample of bonds is required to complete any test of intelligence. Furthermore, different kinds of tests require different samples of bonds. The intercorrelations between tests are caused by an overlap between the bonds tapped by the tests.

In the early to mid-20th century there was a debate between Spearman and Thomson about the interpretation of *g* (see Bartholomew et al. 2009, for a review). Yet, the bonds model has a number of shortcomings (see Eysenck 1987; van der Maas et al. 2006) and, eventually, Spearman’s general ability view was preferred. However, recent simulation studies by Bartholomew and colleagues have shown that sampling is still a viable alternative; from a purely statistical perspective, both the *g* model and the sampling model are sufficient to explain the positive manifold (Bartholomew et al. 2009; Bartholomew et al. 2013).

In addition to specific problems, a major general limitation of the model is that the bonds were left unspecified, that is, they were not interpreted in terms of psychological processes involved in test performance, essentially prohibiting any real-world application of the model. Another problem with the bonds model and sampling models in general is that they fail to capture the hierarchical structure of intelligence. Thomson, like Spearman, proposed that the *g* factor accounts for the covariance among tests. However, according to contemporary models of intelligence, *g* is a higher-order factor and accounts for the observed covariance among broad ability factors, such as verbal and spatial ability. Traditional sampling models do not provide an account of broad ability factors or their relationship to *g*. 

Recent work by Bartholomew and colleagues has sparked a renewed interest in sampling models of *g* (Bartholomew et al. 2009). They updated the original bonds model to show how it can be used to define a subject’s cognitive ability and how it can be used to fit observed data. According to their model, mental capacity is determined by the total number of bonds, such that individuals with more bonds have a greater mental capacity. In their model, it is possible to link bonds to tests, but a limitation of their approach is that there is still no connection between bonds and psychological processes. Arguably, sampling models are of limited value as long as the bonds being sampled are not specified in terms of a psychological theory of test performance.

### 1.2. Process Overlap Theory

Process overlap theory (POT) is a sampling model of *g* that is grounded in psychological theory and supported by evidence from neuroscience (Kovacs and Conway 2016a, 2019a). The fundamental premise of POT is that individual items on tests of intelligence are likely to require multiple cognitive processes, some domain-general and some domain-specific. Crucially, there is no one single process that is required by all items on every test. In other words, there is no general ability; intercorrelations between tests are caused by an overlap between the cognitive processes tapped by the tests. 

As mentioned, POT is largely motivated by cognitive psychology research on working memory. Cognitive models of working memory inform the study of intelligence because working memory capacity is strongly correlated with general intelligence, especially fluid intelligence (Kane et al. 2005). This means that working memory tasks and intelligence tests measure something similar—either the same ability or an overlapping set of abilities. Experimental and correlational investigations of working memory suggest the latter. Experimental evidence is reflected in cognitive models that view working memory as a multicomponent system, consisting of domain-general processes involved in executive attention and domain-specific processes involved in maintenance of information (Baddeley 2000; Cowan 1998; Jonides et al. 2008; Oberauer 2002). Correlational evidence comes from studies of individual differences showing that working memory capacity is not a unitary general factor but instead reflects multiple domain-general abilities (Unsworth et al. 2014), as well as verbal and spatial domain-specific abilities (Kane et al. 2004; Kovacs et al. 2019).

POT, therefore, claims that intelligence, like working memory capacity, is determined by multiple processes. Thus, according to POT, intelligence is best measured by a diverse battery of tests. However, unlike working memory capacity, intelligence is considered to be both domain-general and domain-specific, and some tests are more dependent on domain-general processes while other tests are more dependent upon domain-specific processes. It is the domain-general executive attention processes that are sampled by many different tests, while more specific processes are sampled by a smaller set of tests. 

These central claims are formalized in a multidimensional item response (MIRT) model, which we refer to as POT-I (see Equation (1)).

(1) $$P\left(U_{pi}=1|\Theta_{plm},a_{il},b_{il}\right)=\prod_{l=1}^{D}\frac{e^{\sum_{m=1}^{C}a_{il}\left(\Theta_{plm}-b_{il}\right)}}{1+e^{\sum_{m=1}^{C}a_{il}\left(\Theta_{plm}-b_{il}\right)}}$$

According to POT-I, the probability (*P*) of an individual person (*p*) answering an individual test item (*i*) correctly is a function of their ability level (*Θ*) on the processes required by that item, as well as the discrimination and difficulty parameters for that item. More formally, *Θ_plm_* represents the level of ability for the *p-*th individual on the *m-*th process in the *l-*th domain; *a_il_* is the discrimination parameter for the *i-*th item in the *l-*th domain; *b_il_* is the difficulty parameter for the *i-*th item in the *l-*th domain. *D* is the number of domains sampled by an item, and *C* is the number of processes in a given domain sampled by an item. 

The most important aspect of POT-I is that, as a multidimensional IRT model, it is compensatory within domains and non-compensatory across domains. For instance, if a test requires spatial and executive processes, then a weakness in a spatial process can be compensated for by another spatial process, representing the functioning of these processes as parts of a domain-specific cognitive system, but such compensation cannot take place across domains. Therefore, each domain of a test item functions as a separate dimension, each of which has to be passed independently for the item to be solved correctly. A failure in any one dimension will result in an error, regardless of the level of ability in other domains/dimensions. This aspect of the theory is formalized in POT-I (for a formal introduction to MIRT, see Reckase 2009).

In fact, in addition to specifying actual psychological processes, this is where POT differs most from standard sampling models. Beginning with Thomson (1916), traditional sampling models stressed the random nature of the sampling of processes across items and the processes were left unspecified, i.e., they were not interpreted in terms of psychological processes involved in test performance. Therefore, in standard sampling models, the correlation between any two tests is simply a linear function of the ratio of overlapping and nonoverlapping processes tapped by the tests. In contrast, POT specifies different kinds of processes (spatial, verbal, etc.) and the sampling of processes across items is not random; it is motivated by cognitive psychology research on working memory. Thus, according to POT, the correlation between any two tests is not simply a linear function of the ratio of overlapping and nonoverlapping processes tapped by the tests; instead, correlations between tests are a function of two “sets” of overlapping processes: a set of shared domain-general processes and a set of shared domain-specific processes.

Thus, according to POT-I, overall test performance reflects multiple domain-general abilities and multiple domain-specific abilities (expressed in equation 1 as *Θ_plm_*). This differs from the standard *g* model, motivated by factor analysis, which holds that test performance reflects a single domain-general ability and a single domain-specific ability. As stated earlier, a further claim of POT is that some tests are more dependent on domain-general processes while other tests are more dependent on domain-specific processes. This is the reason why POT accounts for the hierarchical structure of intelligence; it allows for distinct broad ability factors that vary with respect to their relationship to a higher-order general factor.

### 1.3. Theory Building

POT is a united cognitive/psychometric approach to intelligence. More precisely, POT seeks to explain interindividual differences in terms of intraindividual psychological processes. POT is, therefore, uniquely suited to address the question raised by the editors of this Special Issue: What are the executive attention processes underlying *g*? However, POT does not yet explain the relationship between processes and *g*. In order to achieve that level of explanation, we need to engage in a program of research that we refer to as “theory building”. Fried (2020) recently argued that strong theories in the social sciences are lacking and identified some fundamental issues that impede research progress and prevent “theory building”. In our view, the current literature on individual differences in attention and intelligence is plagued by two of the issues raised by Fried: (a) conflation of statistical and theoretical models, and (b) problematic inferences from latent variable models. To be clear, the problems stem from the fact that different researchers have different goals. This section is, therefore, not intended to be a criticism of any one approach; in fact, we focus here on studies by Engle et al. and Miyake et al. because of their strengths. In both of these approaches, the motivation and the goals of the research are clearly articulated, which facilitates the subsequent discussion.

#### 1.3.1. Conflation of Statistical and Theoretical Models 

This is a classic problem in the field of intelligence. Starting with Spearman (1904), psychometric models have been mistaken for psychological theories. In the more contemporary literature, the Cattell–Horn–Carroll model is often referred to as a theory of intelligence. In fact, by some accounts, the CHC *model* is considered to be one the most influential and widely supported “theories” in the field. To be sure, the CHC model is an excellent and well-supported model of the covariance structure of cognitive abilities, and it is compatible with views from developmental and cognitive psychology, but it is not a psychological theory. 

With respect to studies on attention and intelligence, Engle et al.’s line of research on working memory has been extremely influential. Engle et al. take a combined experimental/correlational approach to study individual differences in working memory capacity and attention, which they consider to be domain-general cognitive abilities. The overall approach is often referred to as executive attention “theory” (Engle 2002), which conflates the approach, the psychological theory, and the psychometric model. In our view, it would be more accurate to call it a “framework”. For example, the executive attention *framework* generates theoretically meaningful psychometric *models* to account for individual differences in domain-general cognitive ability. This makes it clear that the goal of this line of research is not to develop a psychological theory of attention. Again, to be clear, the psychometric models developed by Engle et al. are compatible with contemporary psychological theories of attention but the primary focus of the executive attention framework is to develop psychometric models not psychological theories.

Another influential line of research is the work by Miyake et al. on executive functions. Their overall approach is often referred to as the unity/diversity “model” of executive functions. Again, it would be more accurate to call it a “framework”. For example, the goal of the unity/diversity executive attention *framework* is to understand the structure and function of cognitive abilities associated with prefrontal cortex development, damage, and disease, thereby enhancing the assessment, diagnosis, and treatment of cognitive deficits. This allows for a clear distinction between the psychometric model and the psychological theory.

#### 1.3.2. Problematic Inferences from Latent Variable Models

A fundamental difference between Engle et al.’s executive attention framework and POT is that the former considers higher-order reflective factors in psychometric models, such as *g*, to be “psychologically meaningful”, whereas POT does not. Moreover, the studies by Engle et al. are designed to investigate fluid intelligence (Gf), whereas the POT framework was developed to account for both Gf and *g*.

Likewise, a fundamental difference between the unity/diversity framework and POT is that the former considers the higher-order reflective factor in their psychometric model, which is referred to as “common EF”, to be psychologically meaningful, whereas POT does not. Furthermore, Miyake et al. specifically focus on prefrontal functions, which is why they chose to examine “inhibition”, “shifting”, and “updating” (Miyake et al. 2000). Their goal was not to develop a psychometric model of intelligence or to provide a detailed account of *g*. Again, this does not suggest that their model is “wrong”. In fact, the specific focus on cognitive abilities associated with prefrontal damage and disease, which is grounded in neuropsychology, is a unique strength. 

### 1.4. Terminology

Another challenge to theory building, in the context of individual differences in attention, is inconsistent terminology. Attention is a broad topic, relevant in many different subdisciplines of psychology and neuroscience. Unfortunately, the terms used to describe attention are not entirely consistent across fields. Therefore, to avoid confusion, we provide a glossary of common terms in Table 1. It is important to note that these definitions are not intended to be universal; our goal is simply to clarify our own use of these terms, which is influenced by research in cognitive psychology and neuroscience. For each definition in the table, we also provide a reference to work that we consider to be most influential and emblematic of our interpretation.

There is still some lingering ambiguity in the table due to the word “executive”. To clarify, we adopt the following view of “executive processes” from Oberauer (2009): “… not every process executed can be regarded as an executive process without draining the concept executive of all useful meaning. Here, I will make a distinction between the primary processes carrying out the manipulations required for a task, such as mentally moving a pawn on a chessboard, or deciding whether to press the left or the right button in a speeded choice task, and executive processes that supervise and control the primary processes, such as the decision to switch to another task, to update the current WM contents by new information, or to sacrifice accuracy for speed in a choice task.”

Lastly, it is important to note that our definitions of executive attention processes, executive functions, and attention control are intended to map onto different levels of specificity reflected in POT, Miyake et al.’s unity/diversity model, and Engle et al.’s executive attention framework. For example, Engle et al. describe “attentional control” as “a broad and domain-general ability responsible in part for individual differences in most, if not all, higher-order cognitive tasks” (Draheim et al. 2020). Defined in this way, attentional control might consist of multiple executive attention processes, but the number and the nature of processes do not really matter if they are all unitary with respect to individual differences in cognitive ability. Similarly, in the Miyake et al. model, an executive function might consist of multiple executive attention processes, but the number and the nature of those processes do not matter if they do not map onto an established function of the prefrontal cortex that can be identified in neuropsychological assessments. In other words, if a single process is damaged but there is no sign of deficit and no way to assess, diagnose, or treat the damage, then it is irrelevant from the perspective of the executive function model. In contrast, POT is a more theoretical approach, expressed at a different level of specificity, and it would, hence, be relevant to POT.

In our view, this is one of the reasons why research on attention and executive functions is so challenging. The appropriate level of specificity in a model depends on the goals of the researcher. For example, the exact nature of executive functions may be different from a developmental perspective compared to a neuropsychological perspective. The solution to this dilemma is for researchers to be clear about their goals and to clarify their use of terminology. 

### 1.5. Current Studies

Here, we report two studies designed to demonstrate a POT approach to investigate the executive attention processes underlying *g*. The first study is a simulation in which the POT model is used to generate fictional test scores and fictional task measures for a large sample of fictional subjects, more specifically, scores from a large battery of intelligence tests and measures from a set of executive attention tasks (assumed to be relatively process-pure). To preview, the results show that each executive attention measure accounts for unique variance in the general factor obtained from the intelligence test scores. This is essentially a “proof of concept” that POT can provide an account of the executive attention processes underlying *g*. To be clear, a “proof of concept” is simply a demonstration of feasibility; it is not a formal mathematical proof. 

In the second study, we examine the unity/diversity model of executive function (Friedman and Miyake 2017). Specifically, we reanalyze data from three studies with comparable designs to our simulation study, in terms of the intelligence tests and executive attention tasks. To preview, the results show that only one measure of executive function accounts for unique variance in the general factor of intelligence. The results highlight some limitations of previous studies on the unity/diversity model, as well as some advantages of the POT approach. 

## 2. Method, Study 1

The first study is a proof of concept. It is a simulation study designed to show that POT can account for the executive attention processes underlying *g*. For simplicity, we specified just three broad cognitive abilities: verbal, fluid, and spatial. According to the unity/diversity model, we specified three different kinds of executive attention processes: inhibition, updating, and shifting. We also specified some “general” executive attention processes to account for the “unity” aspect of the model, and we included some “non-specified” executive attention processes on the basis of the assumption that there are more than three executive functions.

To obtain a psychometric model of intelligence with a higher-order *g* factor and three manifest indicators of executive attention processes, we generated test scores for three tests of each of the three broad abilities, as well as three tests of executive function (under the assumption of relatively process pure tasks), for a total of 12 tests. Each test consisted of 100 items. Item scores (correct or incorrect; 1 or 0) were based on both domain-specific ability and domain-general ability, which are determined by the specific processes sampled by the item. The sample size was set at *N* = 1000. Lastly, structural equation models (SEM) were used to fit the test score data. The simulation was conducted in R Version 4.0.2 (R Foundation for Statistical Computing, Vienna, Austria) and the SEM analyses were conducted using the “lavaan” package (Rosseel 2012). All R scripts are available online via the Open Science Framework (Hao 2021).

The simulation consisted of four steps: (1) specification of the number and kind of cognitive processes; (2) calculation of domain-general and domain-specific ability parameters (*Θs*); (3) simulation of item responses and calculation of test scores; (4) structural equation model fitting.

### 2.1. Step 1: Specification of Cognitive Processes

For each of the 1000 subjects, 200 cognitive processes were specified. The 200 cognitive processes were classified into four different domains (50 per domain): fluid reasoning processes, verbal processes, spatial processes, and executive attention (EA) processes. The EA processes were subdivided into five types: 10 inhibition processes, 10 updating processes, 10 switching processes, 10 unspecified processes, and 10 common processes. Each subject is thought to have an “ability level” on each of the 200 cognitive processes. These cognitive process abilities are represented by numerical values from a standardized multivariate normal distribution, X_p_ ~ 𝒩_200_ (μ = 0, Σ = I). This simulation step resulted in a 1000 × 200 matrix (number of subjects × number of processes) of orthogonal process ability values. 

### 2.2. Step 2: Calculation of Aggregate Process Ability Parameters (Θs)

The POT model is based on the initial sampling theory by Thomson (1916). A simple elaboration of sampling theory is that the ability, *y* (in this simulation, *Θ*), required for an individual on a specific item of a test can be expressed as

(2) $$y=a^{T}x=\sum_{k=1}^{n}a_{k}x_{k}.$$

In Equation (2), *n* is the number of simulated cognitive processes in the corresponding domain, ***a*** is an *n*-dimensional row vector of random binominal values (0 s and 1 s) for process sampling, and ***x*** is an *n*-dimensional column vector that includes the *n* simulated cognitive processes in this domain for the individual. Therefore, the vector ***a*** expresses how potential processes of the domain are involved for the individual to respond to the item, with *a_k_* = 0 meaning process x_i_ is not sampled and *a_k_* = 1 meaning process *x_k_* is sampled in achieving the corresponding level of ability for the item. As follows, the ratio of 0 s and 1 s in ***a*** vector as the “sampling vector” determines the number of processes being sampled for a *Θ* value.

#### 2.2.1. Tests of Broad Ability 

Each of the nine broad ability tests samples processes from two of the four different domains: one of the three types of domain-specific processes (fluid, verbal, and spatial processes for fluid, verbal, and spatial tests, respectively) plus the domain-general processes (executive attention processes). While executive attention processes were allowed to be sampled in all three types of tests (fluid, verbal, and spatial), the other three types of processes could only be involved in their corresponding test. Hence, for each of the 100 item-level responses of an individual in a test, two aggregate process ability parameters (two *Θs*) were calculated: one domain-general and one domain-specific. The mathematical representation of the POT algorithm in step 2 is described by the following equations (Equations (3) and (4)):

(3) $$\Theta g_{pti}=\sum_{n=1}^{50}b_{tin}G_{pn}$$

(4) $$\Theta s_{pti}=\sum_{m=1}^{50}c_{tim}S_{pm}$$

In these two equations, *Θg_pti_* and *Θs_pti_* are the aggregate domain-general and aggregate domain-specific ability parameters for the *p-*th individual on the *i-*th item of the *t-*th test. The aggregate ability parameters are the sums of sampled processes from their corresponding domains (*G* as the domain-general executive attention processes and *S* as the domain-specific processes) based on the sampling vectors *b_ti_* and *c_ti_.*

An assumption of POT is that all cognitive tests sample domain-general processes and the probability of any one domain-general process being sampled is higher in fluid reasoning tests than in other tests. POT also assumes a large number of cognitive processes per domain (50); thus, the probability of any one process being sampled by a test item is relatively low. In the current simulation, for an item on a domain-specific test (verbal or spatial), the probability of an EA process being sampled was set to 0.12, while the probability of a domain-specific process (verbal or spatial, respectively) being sampled was 0.28, which on average led to the sampling of six executive attention processes and 14 verbal/spatial processes for a domain-specific item. However, for an item on a fluid reasoning test, the probability of an EA process being sampled was set to 0.28, while the probability of a fluid-specific process being sampled was 0.12, which on average led to the sampling of 14 executive attention processes and six fluid processes for an item on a fluid reasoning test. Either way, there were on average a total of 20 processes sampled when generating a simulated response to an item.

#### 2.2.2. Tests of Executive Functions

Each of the three executive function (EF) tests (inhibition, updating, shifting) also sampled processes from two sources, albeit two EA process sources: a task-specific EA source (inhibition, updating, shifting processes) and a general EA source (common executive attention processes). In the simulation, for an item on an EA test, the probability of a specific EA process being sampled was set to 0.28, while the probability of a common EA process being sampled was 0.12, which on average led to the sampling of 2.8 specific EA processes and 1.2 common EA processes. There were on average a total of four processes sampled when generating a simulated response to an item. It is important to note that the pool of EA processes sampled here by the EF tests is the same pool of EA processes sampled by the entire battery of nine cognitive ability tests. In other words, if an EF test sampled EA processes that were not sampled by a battery of cognitive ability tests then we would not expect those EA processes to be associated with *g*. 

Lastly, two 1000 × 12 × 100 (number of subjects × number of tests × number of items per test) three-dimensional arrays of aggregate ability parameters (*Θg* and *Θs)* were created as the result of this step for the broad ability and executive function tests. 

### 2.3. Step 3: Simulation of Item Responses

To generate item-level scores based on POT, a multidimensional IRT function was applied to the domain-general and domain-specific ability parameters (*Θg* and *Θs*). This function is a practical version of the conceptual POT-I model described in Equation (1). The mathematical representation is described by Equation (5).

(5) $$P_{pti}=\sqrt{\frac{1}{1+e^{-\left(z\left(\Theta g_{pti}\right)-bg_{ti}\right)}}\cdot \frac{1}{1+e^{-\left(z\left(\Theta s_{pti}\right)-bs_{ti}\right)}}}$$

where *P_pti_* is the probability of the *p-*th subject getting the *i-*th item in the *t-*th test correct; *z*(*Θg_p_*_ti_) and *z*(*Θs_pti_*) are the standardized scores of *Θg_pti_* and *Θs_pti_,* respectively. In Equation (5), although processes are compensatory within a domain, *Θg* and *Θs* are not compensatory with each other. All discrimination parameters (a parameters) were set to 1 across the 100 items and 12 tests for parsimony. For the nine broad ability tests, the difficulty parameters of the domain-general and the domain-specific processes (*b* parameters, *b_g_* and *b_s_*) for the 900 test items were randomly drawn from a standardized multivariate normal distribution, with each of the nine tests having average *b_g_* and *b_s_* of 0 and standard deviation of 1 and being independent of each other. For the three executive function tests, the difficulty parameters of the domain-general and the domain-specific processes for the 300 test items were also multivariate normal, but with each of the three tests having average *b_g_* and *b_s_* of 0 and standard deviation of 0.05, to reflect the relatively stable performance on EF tasks comparing to broad ability tests. Under this equation the two three-dimensional arrays of *Θg* and *Θs* were transferred to one 1000 × 12 × 100 (number of subjects × number of tests × number of items per test) three-dimensional array of probabilities. A simulated binary response (0 for incorrect response and 1 for correct response) on each item of each test for each individual was generated on the basis of the probabilities from the array. Thus, in general, higher ability would generate a higher probability value to achieve the correct response to the item in a simulation. Overall, for the POT algorithm, this step resulted in a 1000 × 12 × 100 (number of subjects × number of tests × number of items per test) three-dimensional array of binary values (1 and 0), in which 1 represents a correct response and 0 represents an incorrect response.

### 2.4. Step 4: Structural Equation Models

The simulated item-level responses were aggregated per individual, which resulted in a 1000 × 12 (number of subjects × number of tests) test-level dataset. Each subject has one score ranging from 0 to 100 for each of the nine broad ability tests and three executive function tests. We fit two models using confirmatory factor analyses on the generated dataset. The first model was a standard three-factor higher-order model in which the nine manifest variables loaded on three subfactors (fluid, verbal, and spatial), and the subfactors loaded on a higher-order factor (the *g* factor). The second model was the same as the first model with the addition of the inhibition, updating, and shifting tests as predictors of *g*. We refer to these as Model 1 and Model 2 in the results below.

The fit of each model was evaluated with the following set of test statistics and fit indices: χ^2^, comparative fit index (CFI), root-mean-square error of approximation (RMSEA), and standardized root-mean-square residual. The criteria for an “acceptable” model fit were based on the following cutoff values, recommended by (Schreiber et al. 2006): *p* > 0.05, CFI > 0.95, RMSEA < 0.08, SRMR < 0.08.

## 3. Results, Study 1

**Figure 1:**
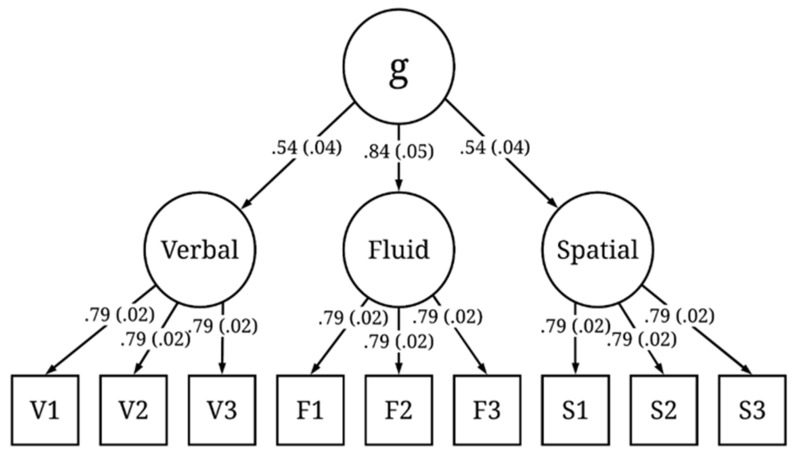
Latent variable model of intelligence based on 200 iterations of simulated test scores. Values represent the mean (and standard deviation) standardized factor loadings.

**Figure 2:**
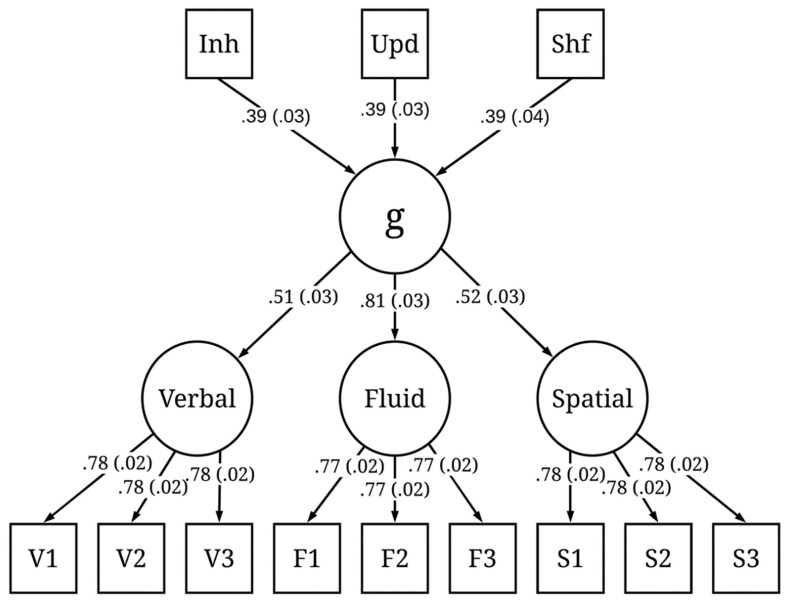
Latent variable model of intelligence and executive attention processes based on 200 iterations of simulated test scores. Values represent the mean (and standard deviation) standardized factor loadings. Inh = inhibition; Upd = updating; Shf = shifting.

We ran 200 iterations of the simulation. The simulated test scores from each iteration were fit to the models presented in Figure 1 and Figure 2. The mean and standard deviation of the standardized factor loadings across the 200 iterations are reported in the figures. Both models exhibited good fit across all iterations. Here, we report the mean fit statistics across 200 iterations. For Model 1, χ^2^(24) = 26.47, *p* = 0.40, CFI = 1.00, RMSEA = 0.01, 90% CI [0.00, 0.03], and SRMR = 0.02. As expected, due to the specified sampling probabilities, the fluid ability factor had a stronger loading on *g* than the spatial or verbal ability factors. For Model 2, χ^2^(51) = 144.59, *p* < 0.001, CFI = 0.98, RMSEA = 0.04, 90% CI [0.03, 0.05], and SRMR = 0.06. Although the χ^2^ statistic was significant, the χ^2^/df ratio was low (χ^2^/df < 3), and the other fit indices all indicated good fit. The three executive attention processes each predicted *g* and each to a similar degree (M = 0.39, SD = 0.03), suggesting that each of these three hypothetical processes explained about 16% of the variance in *g*. Together, the three executive attention processes accounted for 48% of the variance in *g*. It is important to note that, in both Model 1 and Model 2, the three executive attention processes were orthogonal. This was intentional, because, according to POT, there are multiple independent executive attention processes underlying *g* and they each account for unique variance in *g*. 

To further examine the relationship between executive attention processes and *g*, we conducted a series of hierarchical regression analyses to estimate the unique and shared variance in *g* accounted for by each of the processes. This method of analysis, described by Chuah and Maybery (1999), is based on a set of six regression models. For a criterion variable and any three predictors A, B, and C, regression analyses are conducted with the three variables entered in all six possible orders (ABC, ACB, BAC, BCA, CAB, and CBA). One can find the unique contribution of A by finding the *R*^2^ value for a regression that includes A, B, and C and subtracting from it the *R*^2^ value for a regression that includes just B and C; the contribution shared between A and B can be determined by adding the *R*^2^ values for A and B when entered singly and subtracting from that sum the *R*^2^ value for A and B entered together; this continues until all combinations are determined.

**Figure 3:**
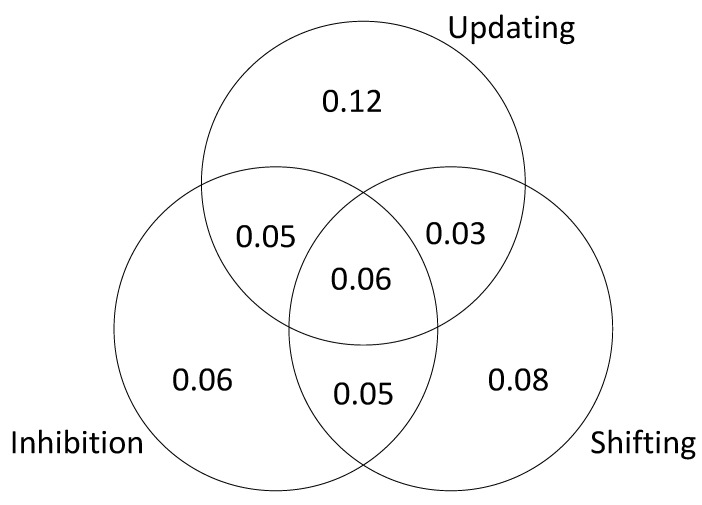
Unique and shared variance in *g* accounted for by executive attention processes.

The results are presented in Figure 3 (based on a single iteration of simulated test scores). The unique variance is due to the fact that we specified different kinds of executive attention processes and then assumed that the tasks successfully isolated those processes. The shared variance is due the fact that we included “common” processes that were sampled by each task. In other words, the tasks were assumed to be relatively process-pure but not completely process-pure. This explains why the results of the SEM are slightly different from the results of the regression analysis; in the SEM models, the executive attention measures were considered to be orthogonal. 

Taken together, these results provide a proof of concept, demonstrating that it is possible for individual differences in multiple executive attention processes to be the underlying source of *g*. To be clear, this is an ideal scenario. In actual studies of individual differences in cognitive ability, the tasks are never process-pure, and there is always some degree of measurement error. The goal in actual research is to design tasks that are as close to process-pure as possible, while producing measures that minimize error and maximize reliability. We contend that, when these conditions are met, it will be possible to conduct a direct test of POT. Specifically, POT predicts that individual differences in attention reflect multiple independent attention “abilities” (executive attention processes) rather than a single unitary attention ability (attentional control). Furthermore, POT predicts that each of the so-called attention abilities accounts for unique variance in *g*. 

## 4. Method, Study 2 

### 4.1. Subjects

Data for a total of *N* = 1422 subjects were analyzed in the current study (Benedek et al. 2014, *N* = 243; Friedman et al. 2006, *N* = 234; Friedman et al. 2011, *N* = 945). Across the three studies, subjects’ mean ages were as follows: Benedek = 23 years, Friedman et al. (2006) = 17 years, Friedman et al. (2011) = 17.3 years. It is important to note that there was some overlap in the Friedman et al. (2006) and Friedman et al. (2011) samples. Friedman et al. (2011) reported that 582 of 945 subjects were included in a study by Friedman et al. (2008). In turn, Friedman et al. (2008) noted that a subsample of their subjects was included in Friedman et al. (2006), but they did not report the exact number. We, therefore, assume that there was some overlap between the 2006 and 2011 samples. However, as noted below, the battery of tasks used to measure intelligence in Friedman et al. (2006) and Friedman et al. (2011) was different. We, therefore, treated the 2006 and 2011 samples as independent despite the fact that there was some overlap. 

### 4.2. Tasks

A summary of the executive attention tasks and intelligence tests (measures of *g*) is provided in Table 2.

### 4.3. Reanalysis Procedure

**Figure 4:**
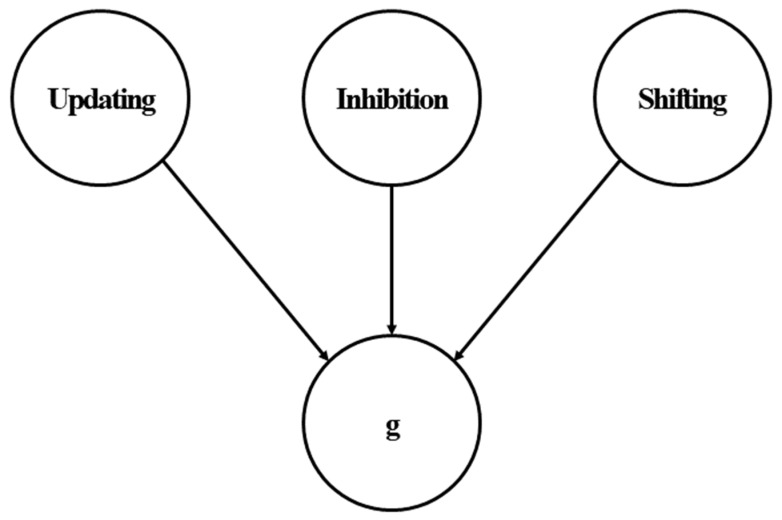
Conceptual model of the relationship between executive functions and *g*.

The purpose of this study was to examine the unity/diversity model of executive functions (Friedman and Miyake 2017). Specifically, we reanalyzed data from three studies with comparable designs to our simulation study, in terms of the intelligence tests and executive attention tasks. This allowed us to specify a psychometric model comparable to the model in Figure 2. For all three datasets (Benedek et al. 2014; Friedman et al. 2006, 2011), the models were specified such that the updating, inhibition, and shifting tasks loaded onto their corresponding latent factors of updating, inhibition, and shifting. The three latent factors of executive functions predicted the *g* factor or the composite score of intelligence tests, depending on the availability of data. The general conceptual model is presented in Figure 4. It is important to note this model was designed for the purposes of the current study, which was motivated by POT. The original studies tested completely different models, which were designed to examine individual differences in a broader range of cognitive abilities, including creativity and self-constraint. As such, the current model results should not be formally compared to the model results reported in the original studies. 

The results of each reanalyzed study should match the results of the simulation study if and only if the tasks are relatively process-pure and the measures are valid indicators of executive attention. Unfortunately, this was not the case. To preview, the results show that only one measure of executive function accounts for unique variance in the general factor of intelligence. Reasons for the discrepancy between the results of the POT simulated model and the results of the unity/diversity model are considered below and in more detail in Section 6. 

For all three datasets, the SEM analyses were conducted in R (Version 4.0.2) using the lavaan package (Rosseel 2012). Correlation matrices obtained from the original publications were used for the analyses. Maximum-likelihood estimation was used for all three SEM models.

The fit indices and criteria were the same as those used in Study 1, i.e., chi-square (χ^2^, nonsignificant χ^2^ indicates good model fit), comparative fit index (CFI, CFI >0.95 indicates good fit), root-mean-square error of approximation (RMSEA, RMSEA <0.08 indicates good fit), and standardized root-mean-square residual (SRMR, SRMR <0.08 indicates good fit). However, it is important to note that the goal of the current study was not to achieve model fit. The goal was to compare models based on real-world data to the model based on simulated data in Study 1. 

## 5. Results, Study 2

**Figure 5:**
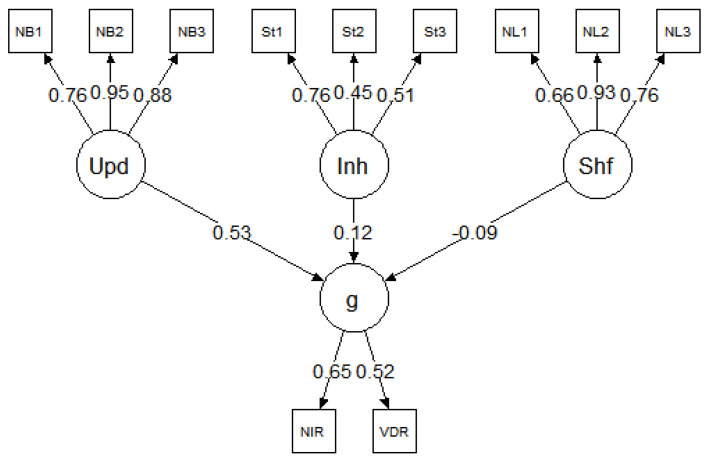
Reanalysis of Benedek et al. (2014). Inh = inhibition; Upd = updating; Shf = shifting; NIR = numerical inductive reasoning; VDR = verbal deductive reasoning.

**Figure 6:**
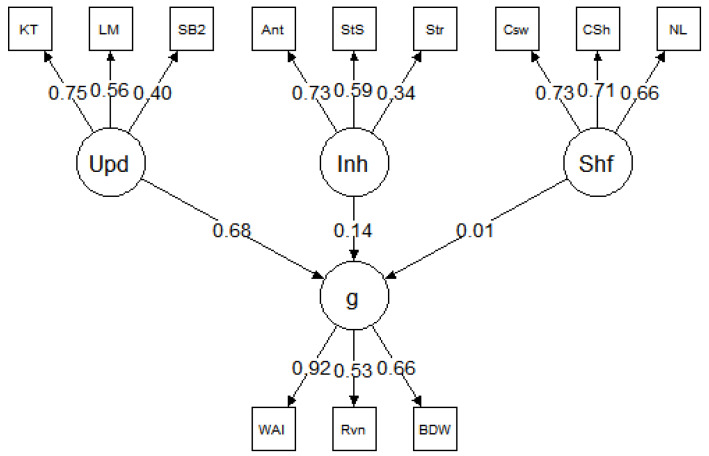
Reanalysis of Friedman et al. (2006). Inh = inhibition; Upd = updating; Shf = shifting; WAI = WAIS IQ; Rvn = Raven’s matrices; BDW = block design WAIS.

**Figure 7:**
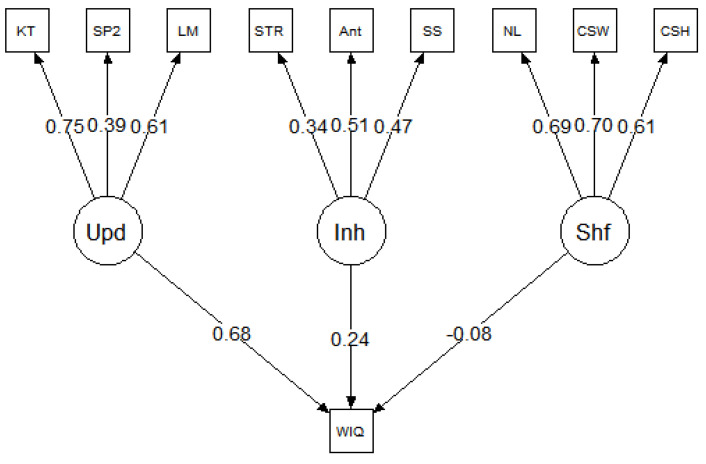
Reanalysis of Friedman et al. (2011). Inh = inhibition; Upd = updating; Shf = shifting; WIQ = WAIS IQ.

The parameter estimates for each model are presented in Figure 5, Figure 6 and Figure 7, and the model fit statistics are reported in Table 3. The results were consistent across datasets; in each model, updating was a strong predictor of *g* but inhibition and shifting were not. Specifically, in all three models, the relationship between updating and *g* was significant: *β* = 0.53 for Benedek et al. (2014), *β* = 0.68 for Friedman et al. (2006), and *β* = 0.68 for Friedman et al. (2011). In contrast, none of the paths between inhibition and *g* or shifting and *g* were significant in any of the models (for all, *p* > 0.05). 

In terms of model fit, the results were somewhat inconsistent across datasets. The Benedek et al. (2014) data fit the model well, as evidenced by a nonsignificant χ^2^ and strong fit indices. In contrast, the Friedman et al. (2006) and Friedman et al. (2011) datasets fit less well. The model χ^2^ statistics were both significant, and all fit indices for both models indicated poor fit. 

**Figure 8:**
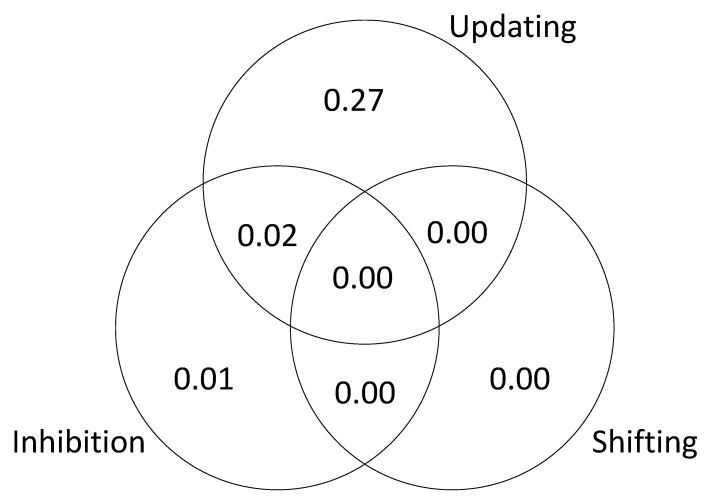
Unique and shared variance in *g* accounted for by executive functions, Benedek et al. (2014).

**Figure 9:**
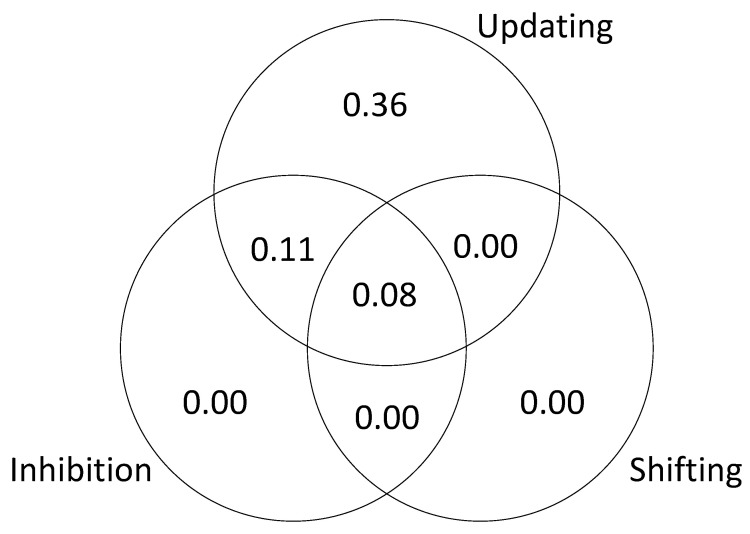
Unique and shared variance in *g* accounted for by executive functions, Friedman et al. (2006).

**Figure 10:**
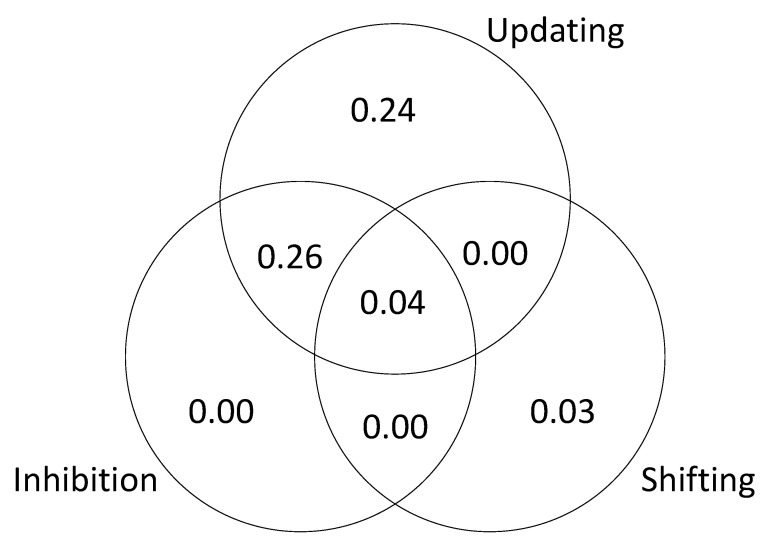
Unique and shared variance in *g* accounted for by executive functions, Friedman et al. (2011).

As in Study 1, to further examine the relationship between executive attention processes and *g*, we tested a series of models to estimate the unique and shared variance in *g* accounted for by each of the processes. The results are presented in Figure 8, Figure 9 and Figure 10. The results of this analysis suggest that the executive function tasks in the studies by Friedman et al. fail to dissociate updating and inhibition (sharing 11% and 26% of explained variance in *g*; see Figure 9 and Figure 10). The results also suggest that shifting does not account for unique variance in *g* (0 to 3% explained variance in *g;* see Figure 8, Figure 9 and Figure 10).

## 6. Discussion

The two studies presented here illustrate a POT approach to investigating individual differences in attention and intelligence. The results of Study 1 provide support for sampling models in general and POT in particular. With respect to sampling models, a general factor (in a higher-order reflective model) emerged from the covariance among test scores *in the absence of a general cognitive ability*. This finding is essentially a proof of concept that sampling is a viable alternative to Spearman’s general ability theory. It is also a replication and extension of the original sampling simulation by Thomson (1916) and the more recent simulation work by Bartholomew et al. (2009). With respect to POT, test scores generated by the POT algorithm fit a higher-order reflective *g* latent variable model, which is the most widely accepted psychometric model in the field of intelligence. This finding provides strong evidence in support of POT. To be clear, this is not *proof* that POT is *right* and general ability theories are *wrong* when it comes to the interpretation of *g*. A general cognitive ability (general intelligence) may or may not exist; however, fitting higher-order models with a causal *g* at the highest level is not the penultimate argument. Such a fit, as we demonstrate here, can be achieved without any kind of general mental ability at play.

The results of Study 1 also support the hypothesis that different executive attention processes account for unique variance in *g*. This result stems from the fact that we assumed the executive attention tasks to be relatively process-pure. That is, the simulation specified different kinds of executive attention processes and each specific task sampled the appropriate kind of process. However, the simulation also included some “general” executive attention processes, which were sampled by all the tasks; thus, they were not assumed to be completely process-pure.

The results of Study 2 reveal differences between the POT approach and the unity/diversity model of executive function (Miyake et al. 2000). Across three datasets, updating accounted for unique variance in *g* but inhibition and shifting did not. The results here are consistent with Friedman and Miyake (2017), but our interpretation is slightly different. Friedman and Miyake (2017) attributed the shared variance between updating and inhibition to a “common” factor, which they interpreted as a cognitive ability. Specifically, they concluded that this common factor “reflects individual differences in the ability to maintain and manage goals, and use those goals to bias ongoing processing”. In our view, the shared variance between updating and inhibition reflects multiple executive attention processes that are sampled by both updating tasks and inhibition tasks. In other words, the tasks are not process-pure. This interpretation is supported by recent individual differences studies in which the cognitive tasks were more carefully designed to isolate specific processes. For instance, recent work has shown that performance on inhibition tasks may be influenced by processing speed and episodic memory, and, in some cases, the variance associated with individual differences in inhibition is task-specific (Rey-Mermet et al. 2019). A separate line of research has revealed that tasks designed to measure updating do not successfully isolate the process of updating; they measure updating, maintenance, and removal of information from the focus of attention (Ecker et al. 2010; Frischkorn et al. 2020). Lastly, we agree with Miyake et al. that goal maintenance is important and plays an essential role in various cognitive tasks; however, in our view, the interpretation of common EF variance as individual differences in goal maintenance is questionable.

This raises the question as to whether it is necessary to isolate specific processes such as goal maintenance, updating, and removal. In our view, it depends on the researcher’s goal. In Miyake et al. (2000), the goal was to identify and measure executive functions, not executive processes. Again, executive functions are defined at a level of specificity that is optimal for researchers in cognitive development and clinical neuropsychology. The fact that a task fails to isolate a cognitive process at lower level of specificity is, therefore, not necessarily a problem. However, if the goal of the research is to identify and measure domain-general executive attention processes at the most specific level possible, then it is a problem. In other words, from the perspective of POT, the shared variance between updating and inhibition is indicative of a measurement problem, which can be addressed in future research, e.g., studies designed to disentangle goal maintenance, updating, and removal. However, from the perspective of the unity/diversity model, the shared variance may have a meaningful interpretation and does not prevent future research on executive function/dysfunction. 

An alternative explanation of the current results is that individual differences in attention reflect a unitary ability, consistent with Engle et al.’ s view of “attentional control”. After all, in the current simulation, domain-general executive attention processes were sampled by every cognitive test. Furthermore, according to the model in Figure 1, *g* is a reflective latent variable, which means that it is the common cause of variance/covariance in the three lower-level factors and, therefore, *reflects* executive attention. Why, then, does POT not equate “attentional control” with *g* and general cognitive ability? First, according to POT, *g* is not a reflective latent variable, it is formative, which means that *g* is an index of overall cognitive ability; it is the consequence, not the cause, of variance/covariance in the lower-level factors. Formative *g*, therefore, represents the aggregate of executive attention processes, verbal ability, spatial ability, and fluid ability. The model in Figure 1 is actually incompatible with POT; it was presented here simply to show that POT can account for a higher-order reflective *g* psychometric structure of intelligence. 

Second, according to POT, there are multiple domain-general executive attention processes. In the current simulation, there were 50. It is, therefore, not necessary to assume that a single process, or ability, is sampled by all tests. More to the point, it is unlikely. At first glance, 50 processes/abilities might seem unrealistic; however, research on individual differences in working memory and cognitive control has already identified several distinct domain-general processes that are related to intelligence: goal activation, goal maintenance, binding, updating, unbinding/removal, controlled retrieval, selective attention, sustained attention, and task switching (Oberauer 2009). Now, if all of these processes were considered to be interchangeable, then it would be fair to interpret the entire set of processes as a unitary general ability. However, a primary goal of our program of research, and a goal of psychological science in general, is to empirically dissociate these kinds of processes, in order to gain insight into things such as cognitive development, learning disabilities, neurological damage and disease, and age-related cognitive decline. The POT framework is specifically designed to facilitate that research effort. 

## Figures and Tables

**Table 1:** Glossary of common terms.

**Cognitive control**	A broad construct that refers to the regulation of information processing during goal-directed behavior. The execution of cognitive control requires executive attention processes, as defined below. The set of processes required depends on the goal, task, context, environment, and individual characteristics. Cognitive control is primarily, but not exclusively, dependent upon the prefrontal cortex and reflects the active maintenance of patterns of neural activity that represent goals and the means to achieve them (Miller and Cohen 2001).
**Attentional control**	A broad cognitive ability that refers to individual differences in cognitive control, as defined above (Draheim et al. 2020).
**Executive function**	A specific cognitive ability that refers to individual differences in cognitive control, as defined above. Functions are more specific than attentional control but more general than executive processes. Functions are defined at a level that is optimal for developmental/neuropsychological assessment, diagnosis, and treatment (Friedman and Miyake 2017).
**Executive process**	A low-level process involved in executive functions, attention control, and cognitive control. Processes are the most specific level in a cognitive model (Oberauer 2009).

**Table 2:** Summary of executive function tasks and intelligence tests analyzed in Study 2.

Study	Inhibition	Updating	Shifting	*g*
**Benedek et al. (2014)**	**Stroop (Block 1):** Subjects were required to respond to the color of presented words on a computer screen. In congruent trials, the color matched the word; in incongruent trials, the color and word did not match. 32 trials total.	**Nonverbal 2-back task (Block 1):** A computer-based task in which abstract figures are presented at a rate of 1.5 s. Subjects are to decide whether the current figure is identical to the one presented two stimuli ago. 33 items per block.	**Number–Letter Task (Block 1):** In this task, subjects are asked to engage in two tasks: determining whether numbers are odd or even and determining whether a letter is a consonant or vowel. 24 trials per block.	**Numerical–inductive reasoning:** An adaptive computerized Subjects are required to determine the rules the govern a series of numbers.
	**Stroop (Block 2):** Same as Block 1.	**Non-verbal 2-back task (Block 2):** Same as Block 1.	**Number-Letter Task (Block 2):** Same as Block 1.	**Verbal-deductive reasoning:** Subjects are required to complete syllogism tasks.
	**Stroop (Block 3):** Same as Block 1.	**Non-verbal 2-back task (Block 3):** Same as Block 1.	**Number-Letter Task (Block 3):** Same as Block 1.	
**Friedman et al. (2006)** **and** **Friedman et al. (2011)**	**Antisaccade:** Subjects suppress a prepotent response to look at a cue and instead look in the opposite direction of the cue.	**Keep-track:** Subjects are presented with a set of 4 categories and a series of 15 words, and then asked to recall the last word presented.	**Number–letter:** Subjects switch between classifying numbers and letters.	**Raven’s Test:** Subjects are to select a tile piece from a set that completes a general complex pattern (used only in Friedman et al. 2006).
	**Stop-Signal:** Subjects are to build a prepotent word categorization response, and then asked to hold the response for trials with beeps.	**Letter memory:** Subjects are presented with a series of letters (lengths: five, seven, or nine letters) and recall the last 3 letters.	**Color–shape:** Subjects shift between classifying shapes and colors.	**WAIS Block Design:** Subjects are to recreate a block design using a given model (used only in Friedman et al. 2006).
	**Stroop Task**: Subjects are to respond to the color font of words and not the word itself.	**Spatial 2-back:** Subjects respond to darkened boxes and decided whether the current box is the same as the one presented two trials prior.	**Category switch:** Subjects shift between classifying the animacy and the size of words.	**WAIS IQ:** A composite general intelligence score calculated from 11 subtests.

**Table 3:** Model fit statistics.

	*χ^2^* (*df*)	*p*	CFI	RMSEA	90% CI	SRMR
Benedek et al. (2014)	41.33 (41)	0.456	1.00	0.01	[0.00, 0.05]	0.05
Friedman et al. (2006)	166.26 (51)	<0.001	0.82	0.10	[0.08, 0.12]	0.14
Friedman et al. (2011)	449.84 (33)	<0.001	0.76	0.12	[0.11, 0.13]	0.15
